# The RAPID-score: Risk Assessment and PredIction of Delirium in acute stroke patients based on very early clinical parameters

**DOI:** 10.3389/fneur.2023.1306520

**Published:** 2023-12-15

**Authors:** Johannes Wischmann, Pauline Kremer, Ludwig Hinske, Roland Tomasi, Andrea S. Becker-Pennrich, Lars Kellert

**Affiliations:** ^1^Department of Neurology, LMU University Hospital, LMU Munich, Munich, Germany; ^2^Department of Anesthesiology, LMU University Hospital, LMU Munich, Munich, Germany; ^3^Institute for Digital Medicine, University Hospital Augsburg, Augsburg, Germany; ^4^Faculty of Medicine, Pettenkofer School of Public Health, Institute for Medical Information Processing, Biometry and Epidemiology (IBE), LMU Munich, Munich, Germany

**Keywords:** delirium, acute stroke, prediction score, stroke unit, intensive care unit, post-stroke delirium

## Abstract

**Background and objective:**

Post-stroke delirium (PSD) is a common complication in acute stroke patients, and guidelines recommend routine screening and various preventive and treatment measures. However, there is a substantial lack of standardized approaches in diagnostic and therapeutic management of PSD. Here, we aimed to develop a new pragmatic and easily assessable screening tool to predict PSD based on early parameters, which are already integral to acute stroke diagnostics.

**Methods:**

We enrolled acute stroke patients admitted to our stroke unit or intensive care unit and developed the scoring system using retrospective single-center patient data. The Confusion Assessment Method for the Intensive Care Unit was used for prospective score validation. Logistic regression models were employed to analyze the association of early clinical and paraclinical parameters with PSD development.

**Results:**

*N* = 525 patients (median age: 76 years; 45.7% female) were enrolled, with 29.7% developing PSD during hospitalization. The resulting score comprises 6 items, including medical history, clinical examination findings, and non-contrast computed tomography results at admission. Scores range from −15 to +15 points, with higher values indicating a higher likelihood of PSD, ranging from 4% to 79%. The accuracy was 0.85, and the area under the curve was 0.89.

**Conclusion:**

The new RAPID (Risk Assessment and PredIction of Delirium in acute stroke patients)-score shows high accuracy in predicting PSD among acute stroke patients and offers precise odds of PSD for each corresponding score value, utilizing routine early clinical and paraclinical parameters. It can identify high-risk populations for clinical study interventions and may be suitable to guide prophylactic PSD measures.

## Introduction

Post-stroke delirium (PSD) is a common complication during hospitalization among patients with acute stroke who are admitted to a stroke unit (SU) or intensive care unit (ICU) (1). It is characterized by attention and concentration disorders, rapid onset, fluctuation throughout the day, and additional cognitive impairments and can manifest as hyperactive, hypoactive, or mixed subtype (2, 3). The detailed pathophysiology of delirium remains unclear; however, it is widely accepted that delirium results from a combination of susceptibility factors and external stressors, including surgery, general anesthesia, or critical illnesses such as stroke (3, 4). The incidence of PSD varies across different studies, with rates ranging from approximately 20 to 35% in SU patients and up to 89% in ICU patients (5, 6). Previous research has shown that PSD is strongly linked to extended hospital stays, ranging from 5 to 22 days longer than stroke patients without PSD. Additionally, it has been found to significantly increase the likelihood of poor functional outcomes, with odds ranging from 2 to 5, and substantially elevate mortality rates, with odds ranging from 2 to 15 times higher depending on the study, stroke subtype, comorbidities and ICU or SU admission (1, 7–12). Several risk factors have been identified, including age, stroke severity, illicit drug use, atrial fibrillation, aphasia, and preexisting dementia (13, 14).

For acute stroke patients, current national and international guidelines recommend regular screening for PSD throughout their hospital stay (15, 16). This can be achieved using tools like the Confusion Assessment Method for the Intensive Care Unit (CAM-ICU) or the Intensive Care Delirium Screening Checklist (ICDSC) (15). In addition, a multidimensional approach for prevention and treatment is recommended, involving both non-pharmacological strategies such as early mobilization, maintaining proper sleep hygiene, and providing reorientation measures, e.g., through the presence of relatives, as well as pharmaceutical approaches, including the use of antipsychotic drugs, particularly in cases of productive psychotic symptoms (15, 17).

Despite being highly relevant in the daily care of acute stroke patients, there is a lack of standardized diagnostic and treatment approaches backed by the highest level of evidence. As a result, the treatment of PSD is often left to the discretion of the treating physicians. Furthermore, the actual clinical practice deviates from the established guidelines. Surveys have revealed that only half of the clinicians have implemented standardized PSD processes, and less than a third regularly employ valid PSD assessments (6, 18, 19). The participants cited a lack of knowledge, staff shortages and insufficient time as the most frequent reasons for this discrepancy. Hence, there is a need for readily accessible and time-saving tools to identify acute stroke patients at a high risk of developing PSD during their hospital stay. This may help tailor and guide prophylactic and therapeutic measures, seamlessly integrating them into daily clinical practice. Our objective was to develop and validate a pragmatic clinical scoring system for predicting PSD in acute stroke patients during hospitalization. The goal was to devise a score based on readily accessible clinical and paraclinical parameters that are already part of the standard diagnostic workup in suspected stroke cases. By doing so, we aimed to facilitate easy implementation without the need for additional screening methods.

## Methods

### Study population and eligibility criteria

**Figure 1:**
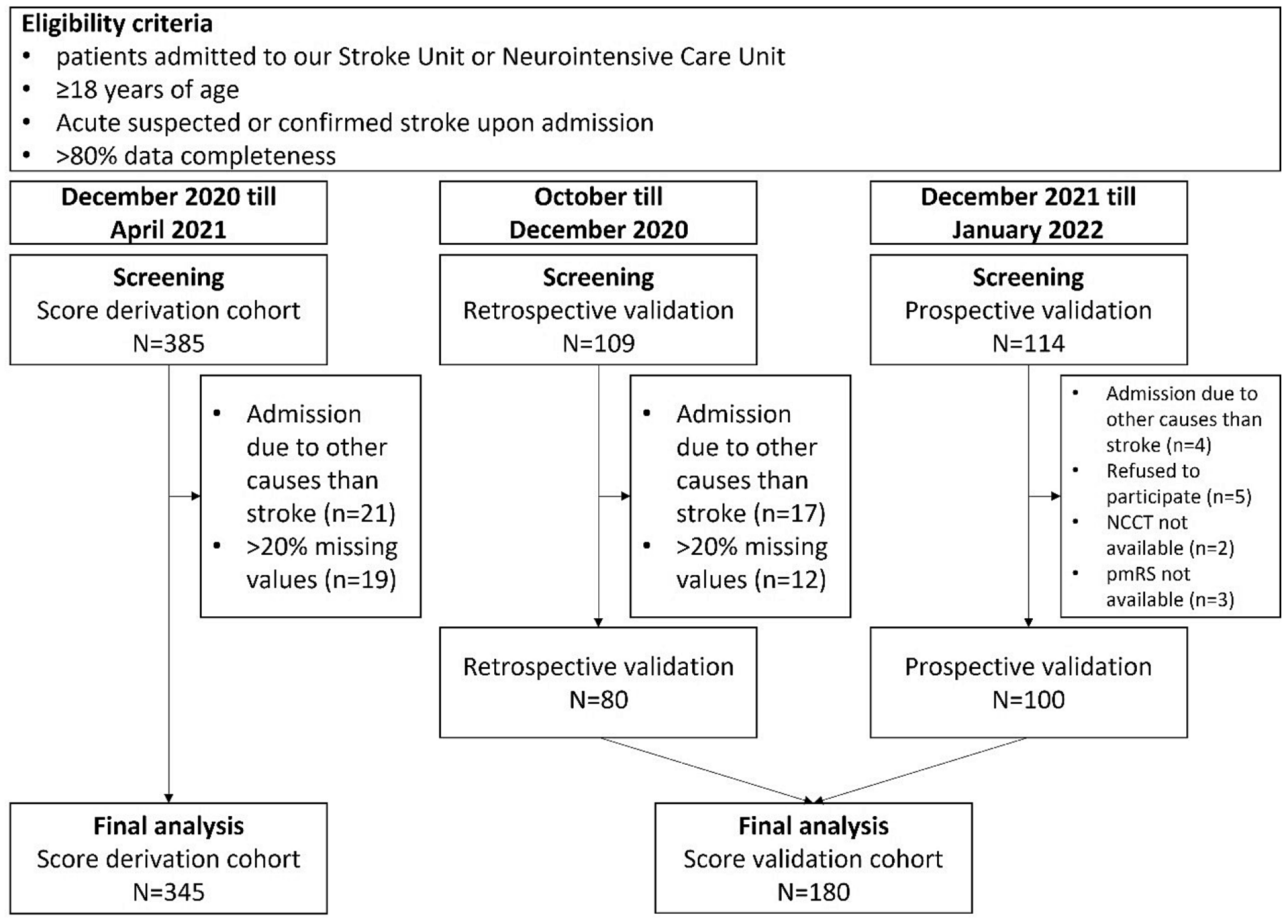
Flowchart for selection and exclusion of patients for final analyses. NCCT, Non contrast computed tomography; pmRS, premodified Rankin Scale.

The study included consecutive patients admitted to our local SU or ICU from October 2020 to April 2021 for score development and retrospective validation. Additionally, consecutive patients admitted between December 2021 and January 2022 were included for prospective validation (Figure 1). The period between May 2021 and November 2021 was used for score development, retrospective validation and preparation of prospective validation. We enrolled all adult patients with confirmed or suspected acute stroke upon admission. All subjects initially underwent evaluation in our emergency department and were subsequently admitted to either the SU or ICU after receiving emergency diagnostics and treatment. A total of *n* = 608 patients were initially screened for the study. Out of these, *n* = 525 patients were enrolled for the final analyses, which included score development and retrospective and prospective score validation. *N* = 388 patients (93.0%) were admitted to our SU, while *n* = 37 patients (7.0%) were referred to ICU upon admission. We excluded *n* = 42 patients who were admitted due to causes other than suspected of confirmed stroke. Additionally, 31 patients were excluded from the retrospective analyses as their data completeness was less than 80%. During the prospective validation phase, *n* = 5 patients refused to provide consent to participate. Moreover, premodified Rankin Scale (pmRS) and non-contrast computed tomography (NCCT) data at admission were missing in *n* = 3 and *n* = 2 patients, respectively in this cohort.

### Score derivation cohort

To develop the scoring system, we retrospectively analyzed consecutive patients (*n* = 345) between December 2020 and April 2021 using electronic medical records. We examined early clinical and paraclinical parameters, including known risk factors for delirium development, that were available after emergency diagnostics and treatment but prior to admission to the SU or ICU. These parameters included age, sex, medical background and history, findings from clinical examinations, vital signs, ECG (electrocardiogram) findings, NCCT scans, CT-angiography (CTA) results, and basic blood examination findings (Table 1). We considered both self-reported information provided by the patients themselves and information provided by others, such as relatives and witnesses, for the medical history and background. Stroke severity and functional status prior to the event were assessed using the National Institutes of Health Stroke Scale (NIHSS) and the pmRS, respectively. Consciousness level was evaluated using the Glasgow Coma Scale (GCS) and the Richmond Agitation-Sedation Scale (RASS). An elevated troponin of >0.015 ng/mL in combination with a creatine kinase (CK)-MB-activity of >24 U/L was defined as myocardial injury. The degree of microangiopathy was rated using the Fazekas score assessed by NCCT (20, 21). All parameters were included in the analysis to predict the occurrence of delirium during the hospital stay. Diagnosis of delirium during the hospital stay was established according to national and international guidelines, such as DSM-5 criteria (22, 23). Detailed assessments of each patient’s medical record were conducted, specifically focusing on the documentation of the patient’s behavior by physicians, nurses, physiotherapists, speech therapists, and occupational therapists. In cases where the diagnosis of delirium was not explicitly documented in the medical records, but there was strong suspicion based on the documented clinical course of the patients (e.g., behavior, necessity for patient restraint, administration of antipsychotic drugs), delirium was presumed for further analysis. Stroke diagnosis at discharge was confirmed by either CT or magnetic resonance imaging. All radiological images were assessed by both radiologists and neuroradiologists as part of the clinical routine, independent from the study.

**Table 1:** Clinical and paraclinical parameters at admission considered for development of the scoring system.

Medical history and comorbidities	Age, sex, pmRS, oral anticoagulation, history of epilepsy, any neurodegenerative comorbidity, any psychiatric comorbidity, arterial hypertension, diabetes mellitus, hyperlipidemia, atrial fibrillation, cancer diagnosis, cerebral metastasis, traumatic brain injury or stroke
Vital signs at admission	systolic blood pressure, diastolic blood pressure, heartrate, peripheral capillary oxygen saturation, body temperature
Clinical examination	NIHSS, GCS and RASS at admission
ECG at admission	Atrial fibrillation
Blood examination	Sodium, potassium, creatine kinase, myocardial injury, C-reactive protein, hemoglobin, hematocrit, GOT, GPT, LDH, leucocytes, Quick, INR, TSH
Non-contrast computed tomography	ICH, SAH, epidural hemorrhage, subdural hemorrhage, other intracranial space-occupying lesion, infarct demarcation, Fazekas-Score, global brain atrophy
Computed tomography angiography	Anterior circulation vessel occlusion, posterior circulation vessel occlusion, cerebral venous thrombosis, aneurysm

PmRS, premodified Rankin Scale; NIHSS, National Institutes of Health Stroke Scale; GCS, Glasgow Coma Scale; RASS, Richmond Agitation Sedation Scale; ECG, electrocardiogram; GOT, glutamic-oxaloacetic transaminase; GPT, glutamic-pyruvic transaminase; LDH, Lactate dehydrogenase; INR, International Normalized Ratio; TSH, thyroid stimulation hormone; ICH, intracranial hemorrhage; SAH, subarachnoid hemorrhage.

### Score validation cohort

To prospectively validate the scoring system, we enrolled eligible patients (*n* = 100) consecutively admitted to either our SU or ICU between December 2021 and January 2022. Written informed consent was obtained from patients or from their legal representatives in cases where patients were unable to provide consent. In situations where patients initially lacked the capacity to consent but later regained it, informed consent was obtained retrospectively directly from the patients themselves. All variables listed in Table 1 were assessed for each patient. Delirium was assessed employing the DSM-5 criteria and additionally using the CAM-ICU (8, 24, 25) immediately upon admission, as well as at the 24-h and 72-h marks after admission. Radiologists and neuroradiologists conducted assessments of all radiological images without knowledge of the subject’s delirium status, both at the time of imaging and throughout the clinical course. For internal retrospective validation, we analyzed electronic medical records of *n* = 80 consecutive patients between October 2020 and December 2020 who were separate from those included in the score derivation cohort.

## Statistical analysis

Statistical analysis was conducted in Python 3.8.0 with Jupyter Notebooks 6.2.0. The following libraries were used: numpy (version 1.19.5), pandas (1.2.0), matplotlib (3.4.3), seaborn (0.11.1), scipy (1.6.0) and sklearn (0.24.1). The random state was set to 42 and used whenever applicable. We excluded columns with only one unique value. Missing values were imputed using an iterative imputer with 10 iterations. All columns with continuous data were categorized using the 33rd and 66th percentile: less or equal than the 33rd percentile, from 33rd to less than or equal the 66th percentile, or greater than the 66th percentile. In the case of the 33rd percentile being the same as the 66th percentile, we binarized the variable by the maximum value or less. Categorical variables were dummy-coded. A cross-validated logistic regression with 100,000 maximum iterations, a L2 penalty term and a liblinear solver were used for a 5-fold cross-validated recursive feature elimination, using accuracy as the performance metric. The minimum number of features to select was set to three. The number of features corresponding to the first local maximum of accuracy was then selected. A recursive feature elimination using the same logistic regression with the selected number of features was used to identify the parameters used for the following score development. All labels and the selected features were split into a training and a test set with a test size of 0.3. The training set was used to train a cross-validated logistic regression with a maximum of 50,000 iterations, a L2 penalty term and a liblinear solver. For the score development, we scaled all coefficients using $\mathrm{factor}=\frac{20}{\ln\left(20\right)}$ and rounded these numbers to the nearest whole number. The positive predictive value, negative predictive value, sensitivity, specificity, accuracy score and AUC ROC score were calculated on the test set. The goodness of fit was tested using the Hosmer-Lemeshow test. The chi-square test of independence was used to test categorical variables between two groups, the Mann–Whitney U test was used to test for differences between continuous variables between two groups. An alpha-level of 0.05 was considered statistically significant. Univariate analysis was conducted, comparing parameters between patients with and without development of delirium during hospital stay. Each variable is displayed with median and interquartile range (IQR) or counts and percentages, where applicable.

## Results

### Demographics and descriptive statistics

In total, *n* = 525 patients (median age: 76 years; 45.7% female) were enrolled into final analysis. *N* = 156 (29.7%) developed delirium during hospitalization and suspected stroke was confirmed in *n* = 330 patients (62.9%) at discharge. In the score derivation cohort (*n* = 345; 46.1% female; Table 2), *n* = 98 patients (28.4%) developed delirium during their hospital stay, while in the score validation cohort (*n* = 180; 45.0% female), *n* = 58 (32.2%) patients experienced delirium during hospitalization. While there was no significant difference in the rate of confirmed stroke diagnosis at discharge in the derivation cohort, patients with delirium did experience longer hospital stays (8 vs. 5 days). They were significantly older (80 vs. 73 years) and upon admission, these patients exhibited higher pmRS-Scores (3 vs. 0) and were more likely to have pre-existing medical conditions, including a history of epilepsy (13.3 vs. 4.5%), cancer (31.6 vs. 14.6%), stroke (29.6 vs. 16.2%), neurodegenerative comorbidities (17.3 vs. 2.8%), and atrial fibrillation (32.7 vs. 11.3%). Additionally, a greater number of them were under oral anticoagulation upon admission (31.6 vs. 12.6%) and atrial fibrillation was more frequently detected in the initial admission ECG (33.7 vs. 9.7%). Moreover, patients who developed delirium during their hospital stay more often presented with impaired consciousness and more severe strokes, as indicated by lower GCS (14 vs. 15 points) and RASS scores, while their NIHSS scores were higher (7 vs. 2 points). One relevant distinction in laboratory findings was observed, with a higher rate of myocardial injury in the delirium cohort (20.4 vs. 6.1%). Furthermore, the NCCT at admission more frequently revealed the presence of microangiopathy (defined by a Fazekas-Score ≥ 1 point; 91.8 vs. 49.8%) and global brain atrophy (55.1 vs. 17.4%).

**Table 2:** Descriptive analyses of the derivation cohort.

Clinical and paraclinical parameters	Delirium (*n*=98)	No delirium (*n*=247)	*p*-value
**Age (years), median (IQR)**	**80 (74–84)**	**73 (58–80)**	**<0.001**
Female sex, *n* (%)	42 (42.9)	117 (47.4)	0.523
Stroke diagnosis at discharge, *n* (%)	52 (53.1)	156 (63.2)	0.108
**Length of stay (days), median (IQR)**	**8 (4–13)**	**5 (2–8)**	**<0.001**
**Medical history**			
**PmRS, median (IQR)**	**3 (1–5)**	**0 (0–0)**	**<0.001**
**Epilepsy, *n* (%)**	**13 (13.3)**	**11 (4.5)**	**0.008**
**Neurodegenerative comorbidity, *n* (%)**	**17 (17.3)**	**7 (2.8)**	**0.015**
Psychiatric comorbidity, *n* (%)	14 (14.3)	19 (7.7)	0.094
**Previous stroke, *n* (%)**	**29 (29.6)**	**40 (16.2)**	**0.008**
Previous TBI, *n* (%)	7 (7.1)	8 (3.2)	0.097
Arterial hypertension, *n* (%)	64 (65.3)	152 (61.5)	0.597
Diabetes, *n* (%)	20 (20.4)	36 (14.6)	0.245
Hyperlipidemia, *n* (%)	18 (18.4)	39 (15.8)	0.674
**Atrial fibrillation, *n* (%)**	**32 (32.7)**	**28 (11.3)**	**0.012**
**Oral anticoagulation, *n* (%)**	**31 (31.6)**	**31 (12.6)**	**0.041**
Cerebral metastasis, *n* (%)	1 (1.0)	3 (1.2)	0.685
**Cancer, *n* (%)**	**31 (31.6)**	**36 (14.6)**	**<0.001**
**Clinical examination**			
**GCS at admission, median (IQR)**	**14 (12–15)**	**15 (15–15)**	**<0.001**
**NIHSS at admission, median (IQR)**	**7 (3–12)**	**2 (0–6)**	**<0.001**
**RASS at admission, median (IQR)**	**0 [(−1)–0]**	**0 (0–0)**	**0.041**
**Vital signs at admission, median (IQR)**			
BP systolic (mmHg)	177 (159–220)	171 (156–270)	0.404
**BP diastolic (mmHg)**	**85 (74–96)**	**90 (79–98)**	**0.023**
Heartrate (per minute)	83 (70–95)	80 (70–90)	0.181
Body temperature (°C)	36.9 (36.6–39.1)	36.8 (36.6–38.9)	0.219
Peripheral capillary oxygen saturation (%)	99 (96–100)	97 (95–98)	0.483
**Electrocardiogram at admission**			
**Atrial fibrillation**	**33 (33.7)**	**24 (9.7)**	**<0.001**
**Laboratory parameters**			
Sodium (mmol/L), median (IQR)	140 (137–142)	139 (137–141)	0.410
Potassium (mmol/L), median (IQR)	4.3 (4–4.6)	4.3 (4.1–4.6)	0.274
Creatine kinase (IU/mL), median (IQR)	94 (54–157)	97 (67–139)	0.264
**C-reactive protein (mg/dL), median (IQR)**	**0.5 (0.2–1.9)**	**0.2 (0.1–0.7)**	**0.009**
**Myocardial injury, *n* (%)**	**20 (20.4)**	**15 (6.1)**	**<0.001**
**GOT (IU/L), median (IQR)**	**26 (19–33)**	**21 (17–27)**	**0.001**
**GPT (IU/L), median (IQR)**	**18 (13–25)**	**20 (15–28)**	**0.019**
**LDH (IU/L), median (IQR)**	**232 (183–283)**	**192 (167–237)**	**0.030**
**Hemoglobin (g/L), median (IQR)**	**13.0 (11.3–14.2)**	**14.0 (12.6–15.1)**	**0.023**
**Hematocrit (%), median (IQR)**	**0.38 (0.34–0.41)**	**40.7 (37.2–43.8)**	**0.028**
**Leucocytes (g/L), median (IQR)**	**8.7 (7.1–12.0)**	**7.9 (6.4–10.3)**	**0.008**
**Quick (%), median (IQR)**	**103 (88–114)**	**112 (101–12)**	**0.007**
**INR, median (IQR)**	**1.0 (0.9–1.1)**	**0.9 (0.9–1.0)**	**0.007**
TSH (mU/L), median (IQR)	1.48 (0.79–2.42)	1.58 (1.01–2.29)	0.250
**Computed tomography imaging**			
Left hemispheric lesion, *n* (%)	23 (23.5)	55 (22.3)	0.468
Infarct demarcation, *n* (%)	17 (17.3)	40 (16.2)	0.921
Hyperdense media sign, *n* (%)	4 (4.1)	14 (5.7)	0.742
ICH, *n* (%)	8 (8.2)	21 (8.5)	0.910
SAH, *n* (%)	(2.0)	8 (3.2)	0.809
Epidural hemorrhage, *n* (%)	0 (0.0)	0 (0.0)	0.348
Subdural hemorrhage, *n* (%)	4 (4.1)	3 (1.2)	0.201
Other intracranial space-occupying lesion, *n* (%)	2 (2.0)	8 (3.2)	0.809
**Fazekas-score ≥1, *n* (%)**	**90 (91.8)**	**123(49.8)**	**<0.001**
**Fazekas-Score, median (IQR)**	**2 (1–3)**	**0 (0–2)**	**<0.001**
**Global brain atrophy, *n* (%)**	**54 (55.1)**	**43 (17.4)**	**<0.001**
Anterior circulation vessel occlusion, *n* (%)	16 (16.3)	43 (17.4)	0.192
Posterior circulation vessel occlusion, *n* (%)	6 (6.1)	12 (4.9)	0.835
Aneurysm, *n* (%)	2 (2.0)	10 (4.0)	0.554
Central venous thrombosis, *n* (%)	0 (0.0)	0 (0.0)	0.271

PmRS, premodified Rankin Scale; GCS, Glasgow Coma Scale; NIHSS, National Institutes of Health Stroke Scale; RASS Richmond Agitation Sedation Scale; BP blood pressure; GOT, glutamic-oxaloacetic transaminase; GPT, glutamic-pyruvic transaminase; LDH, Lactate Dehydrogenase; INR, International Normalized Ratio; TSH, thyroid stimulation hormone; ICH, intracranial hemorrhage; SAH, subarachnoid hemorrhage. Differences among both groups are marked bold.

### The score development

All parameters were taken into account for score development, except for “epidural hemorrhage in NCCT,” as this condition was not present in any of the patients in our cohort. Among the CTA variables, there were 37 missing values, while thyroid stimulating hormone had 29 missing values, and 24 other variables had a much lower extent of missing data (Supplementary Table). After imputing the missing data, the dataset was transformed and categorized, resulting in a total of 94 parameters for further analysis. The cross-validated recursive feature elimination returned 90 features to be the optimal number (accuracy = 0.7913). The first local maximum of accuracy scores was found for the amount of seven features (0.7101), which was used in a recursive feature elimination to identify the items used for the scoring system. The seventh feature, an INR of ≤0.9, predicted the occurrence of delirium. However, this particular feature was not included in the subsequent score development due to concerns related to clinical plausibility and practicality. The points for each score item were calculated based on the coefficients of the logistic regression.

### The scoring system

**Figure 2:**
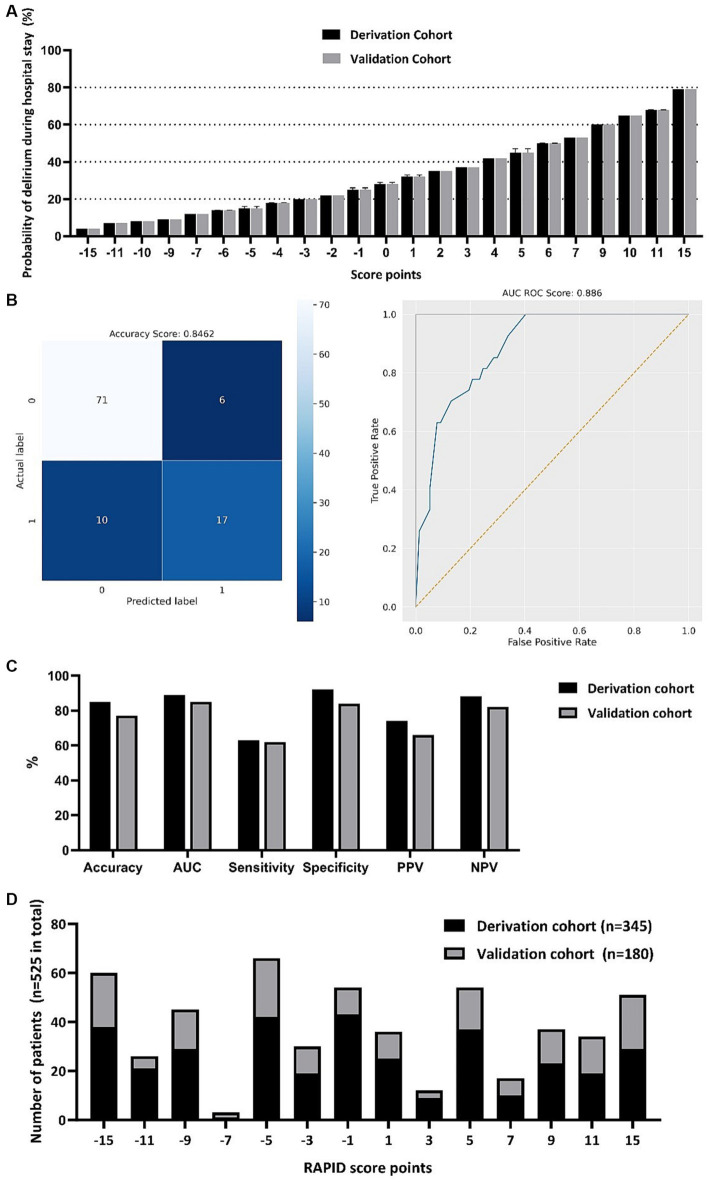
RAPID-score points with corresponding probability of developing delirium during the hospital stay **(A)**. Computed values are depicted for both the derivation and the validation cohort with mean, minimum and maximum. Confusion matrix of the scoring system and receiver operation characteristic (ROC) curve in the score derivation cohort **(B)**. Score performance value displayed for the score derivation and validation cohort **(C)**. Frequency distribution of total RAPID-score points in both the derivation and validation cohort **(D)**. AUC, Area under the curve; PPV, positive predictive value; NPV, negative predictive value.

The scoring system, which we named **RAPID** (**R**isk **A**ssessment and **P**red**I**ction of **D**elirium in acute stroke patients; Table 3) consists of 6 items and covers a score range from −15 to +15 points. Higher scores on this system indicate a greater likelihood of experiencing delirium during the hospital stay (Figure 2A). A GCS score of <15 at admission adds 6 points, a Fazekas Score ≥ 1 in NCCT at admission adds 5 points, and the presence of global brain atrophy in NCCT contributes 4 points to the total score. On the other hand, being aged ≤68 years deducts 4 points, a NIHSS score ≤ 1 subtracts 5 points, and a pmRS score of 0 takes away 6 points from the total score.

**Table 3:** The **RAPID**-score (**R**isk **A**ssessment and **P**red**I**ction of **D**elirium in acute stroke patients).

Score item	Points	Coefficient	Odds ratio	Factor
Glascow Coma Scale <15 points at admission	+6	0.9375	2.5535	6.2687
Fazekas Score ≥ 1 point in non-contrast computed tomography	+5	0.7424	2.101	4.9564
Global brain atrophy in non-contrast computed tomography	+4	0.6404	1.8972	4.2753
Age ≤ 68 years	−4	−0.5856	0.5568	−3.909
National Institutes of Health Stroke Scale ≤1 point	−5	−0.7354	0.4793	−4.910
Premodified Rankin Scale =0 points	−6	−0.9222	0.3976	−6.1567

Each score item is presented with its corresponding score points contributing to the total score, the coefficient from the logistic regression analysis, the odds ratio of developing delirium during hospital stay for each item, and the respective contributing score factor.

### Score performance and validation

In the derivation cohort, we achieved a computed accuracy of 0.8462 and an area under the curve (AUC) of 0.8860 (Figure 2B). For the validation cohort, the accuracy and AUC were slightly lower at 0.7722 and 0.8498, respectively. Regarding the positive predictive value (PPV) and negative predictive value (NPV), in the derivation cohort, we obtained a PPV of 0.7391 and an NPV of 0.8765. In the validation cohort, the PPV was 0.6545, and the NPV was 0.8220. We found that in the derivation cohort, the sensitivity was 0.6296, and the specificity was 0.9221. In the validation cohort, the sensitivity and specificity were 0.6207 and 0.8443, respectively (Figure 2C). The median RAPID score was −1 in both the derivation and validation cohorts, indicating a 25% probability of delirium development during the hospital stay. The frequency distribution is depicted in Figure 2D.

## Discussion

The RAPID-score is a pragmatic clinical screening tool with high accuracy in predicting PSD during the hospital stay of acute ischemic stroke patients. This accuracy was consistent in both the score development and validation cohorts. It incorporates readily available parameters, such as medical history, clinical examination, and NCCT findings taken upon admission, all of which are integral to basic stroke diagnostics and already obtained during emergency department processing. In fact, the RAPID-score enables the prediction of delirium and risk assessment within the first hour after emergency admission of the patient. Furthermore, each RAPID-score point is linked to a precise probability of developing delirium during hospitalization, and this association shows minimal variance among the cohorts included in our study. Its seamless integration into clinical routine makes it easily adaptable, however the RAPID-Score does not aim to replace regular delirium screening during the hospital stay, using the CAM-ICU or ICDSC, as recommended by current guidelines. Instead, it may be a complementary tool for identifying high-risk populations, guiding prophylactic measures, or identifying candidates for future study interventions.

We observed delirium in approximately 30% of the patients, a rate comparable to existing data, considering that our study also included ICU patients, where delirium rates tend to be higher than in SU patients (26). Consistent with previous findings, we identified several factors predicting PSD, including older age, impaired consciousness, and the severity of strokes, as indicated by higher NIHSS scores (14, 27). Furthermore, we conducted univariate analyses for differences in single parameters between delirium and no-delirium patients. Here, we also observed higher rates of preexisting medical conditions in patients who developed PSD, such as atrial fibrillation, previous strokes, epilepsy, cancer, and neurodegenerative comorbidities, which most likely translate into higher pmRS scores in those patients. Moreover, cerebral microangiopathy and global brain atrophy, assessed by NCCT, turned out to be strong predictors for delirium within the scoring system, likely correlating with observed higher rates of preexisting neurodegenerative diseases, such as dementia, which have also previously been associated with an increased risk of delirium (7). However, the association of white matter lesions in acute stroke imaging and post-stroke cognitive decline several months after stroke onset remains subject to ongoing discussions (16). Interestingly, we found that none of the parameters assessed by NCCT and CTA at admission, which define stroke type (e.g., rate of intracerebral hemorrhage, infarction demarcation and side, and type of vessel occlusion), differed significantly between patients with and without PSD during their hospital stay. This suggests that the observed higher NIHSS scores in patients with delirium may likely not solely attributed to more severe stroke types but also to their higher susceptibility to stroke due to preexisting medical conditions.

Similar to other post-stroke prediction scores, our final score incorporated age and stroke severity. However, unlike those scores that only included stroke patients, we did not find a significant association between PSD and specific stroke subtypes (e.g., ICH, anterior vessel occlusion) or any laboratory markers, which could be attributed to the broader inclusion of patients with stroke mimics in our study (28–30). Regarding accuracy, sensitivity, and specificity, our score demonstrated comparable results to those of other prediction scores, however some prediction scores published elsewhere lack external validation and have smaller sample sizes, potentially affecting their generalizability and reliability (16, 28, 29). Moreover, when compared to other scoring systems, our presented score not only offers cut-off values but also provides precise probabilities of PSD development associated with each corresponding score value.

One of the key strengths of our study is the utilization of a relatively large sample size of well-characterized patients with high data completeness. Moreover, the score underwent validation in both retrospective and prospective independent cohorts. Notably, we have included stroke mimics in the study, enhancing the clinical relevance of our findings as it reflects real-world scenarios. Approximately one-third of our enrolled patients were identified as stroke mimics, with infectious causes, peripheral vestibular disorders and seizure attacks being the most common mimic, consistent with previous literature (31). However, inclusion of stroke mimics might have limited sensitivity and specificity, due to increased patient heterogeneity. Despite this, it has improved the generalizability of our results. Interestingly, we observed comparable rates of delirium during hospitalization in patients with stroke mimics, when compared to patients with confirmed strokes, underlining the importance of conducting delirium risk assessments in SU or ICU patients regardless of the specific diagnosis. The primary limitation of our study is the likely underdiagnosis of hypoactive delirium and the differentiation of pre-stroke cognitive decline, delirium and stroke-related cognitive deterioration independent from delirium, which is however a common limitation encountered in delirium assessments and applies to our study as well (3, 32, 33). Furthermore, the CAM-ICU has its weaknesses when applied in stroke patients and is not exactly equivalent to delirium assessment according to DSM-5 criteria (34, 35). Moreover, it is well known, that aphasia, preexisting cognitive impairment and dementia leads to diagnostic uncertainty in delirium assessment. Approximately 14% of our patients exhibited stroke-related aphasia, while 7% of our subjects had a preexisting neurodegenerative comorbidity. These factors may have influenced our findings and could have implications for the accuracy of our results and score performance. Additionally, since the score relies on information from the patient’s medical history, it may not be immediately applicable upon admission in cases where the patient is unable to provide this information independently, and no relatives are present. Furthermore, as our objective was to pragmatically assess scores immediately after patient admission, we only considered factors readily available early in the admission process for score development. Consequently, we cannot dismiss the possibility of overlooking other significant predictors for the development of PSD later in the patient’s clinical course.

## Conclusion

The newly introduced RAPID-score is able to predict PSD among acute stroke patients with high accuracy. It incorporates early clinical and paraclinical parameters that are already integral to the standard diagnostic workup for suspected stroke cases, making it broadly adoptable and time efficient. It can identify high-risk individuals for clinical study interventions, and may be used to devise targeted strategies for the prevention of PSD.

## Data availability statement

The original contributions presented in the study are included in the article/Supplementary material, further inquiries can be directed to the corresponding author.

## Ethics statement

The studies involving humans were approved by Ethics Committee of the LMU Munich, LMU University Hospital Munich, Germany. The studies were conducted in accordance with the local legislation and institutional requirements. The participants provided their written informed consent to participate in this study.

## Author contributions

JW: Conceptualization, Formal analysis, Investigation, Methodology, Project administration, Supervision, Validation, Visualization, Writing – original draft, Writing – review & editing. PK: Conceptualization, Data curation, Investigation, Methodology, Writing – review & editing. LH: Conceptualization, Formal analysis, Investigation, Methodology, Supervision, Validation, Writing – review & editing. RT: Conceptualization, Formal analysis, Investigation, Methodology, Supervision, Validation, Writing – review & editing. AB-P: Conceptualization, Formal analysis, Investigation, Methodology, Validation, Writing – original draft, Writing – review & editing. LK: Conceptualization, Investigation, Methodology, Project administration, Resources, Supervision, Validation, Writing – review & editing.
