# Supplementary material for: The RAPID-score: Risk Assessment and PredIction of Delirium in acute stroke patients based on very early clinical parameters
Source: Front Neurol. 2023 Dec 15;14:1306520. doi: 10.3389/fneur.2023.1306520 (PMC10756062; doi:10.3389/fneur.2023.1306520)
Supplement: Supplementary file 1 [file Table_1.DOCX]

**Supplemental Table**

| **Variables** | **Missing values (n)** |
| --- | --- |
| **Categorized (yes/no)** | |
| Anterior circulation vessel occlusion | 37 |
| Posterior circulation vessel occlusion | 37 |
| Aneurysm | 37 |
| Atrial fibrillation (initial ECG) | 7 |
| Hyperlipidemia | 2 |
| Oral anticoagulation | 2 |
| Global brain atrophy | 2 |
| Arterial hypertension | 1 |
| Hyperdense media sign | 1 |
| **Continuous, median (IQR)** | |
| Thyroid stimulating hormone | 29 |
| Lactatdehydrogenase | 10 |
| Premodified Rankin Scale | 9 |
| Glutamic oxaloacetic transaminase | 6 |
| Glutamic pyruvic transaminase | 6 |
| National Institutes of Health Stroke Scale | 4 |
| Body temperature | 3 |
| Quick | 2 |
| Richmond Agitation Sedation Scale at admission | 1 |
| Systolic blood pressure at admission | 1 |
| Diastolic blood pressure at admission | 1 |
| Heartrate | 1 |
| Peripheral capillary oxygen saturation | 1 |
| Creatine kinase | 1 |
| Hematocrit | 1 |
| Fazekas-Score | 1 |
